# Factors Influencing Antibiotic Prescribing and Antibiotic Resistance Awareness Among Primary Care Physicians in Poland

**DOI:** 10.3390/antibiotics14020212

**Published:** 2025-02-19

**Authors:** Karolina Świder, Mateusz Babicki, Aleksander Biesiada, Monika Suszko, Agnieszka Mastalerz-Migas, Karolina Kłoda

**Affiliations:** 1 NZOZ Biogenes, 53-224 Wroclaw, Poland; karolina.swider.pl@gmail.com; 2 Department of Family Medicine, Wroclaw Medical University, 50-367 Wroclaw, Poland; agnieszka.mastalerz-migas@umw.edu.pl; 3 Polish Society of Family Medicine, Ul. Syrokomli, 51-141 Wroclaw, Poland; alek.biesiada@gmail.com; 4 Department of General Surgery and Clinical Nutrition, Medical Center of Postgraduate Education Warsaw, 01-813 Warsaw, Poland; msuszko@cmkp.edu.pl; 5 MEDFIT Karolina Kłoda, Ul. Narutowicza, 70-240 Szczecin, Poland; wikarla@gazeta.pl

**Keywords:** antibiotics, primary care, antibiotic prescribing, antibiotic resistance

## Abstract

**Introduction**: Antibiotic resistance is a major public health problem in Europe. Most antibiotics are sold only by prescription in Poland, and it is mainly up to physicians to decide whether to start antibiotic treatment. Therefore, we analyzed the factors influencing the prescribing of antibiotics for upper respiratory tract infections by primary care physicians in Poland, attitudes toward antibiotic resistance, and knowledge of the principles of antibiotic use. **Methods**: We conducted a CAWI (Computer-Assisted Web Interview) survey, carried out using a proprietary survey distributed online. **Results**: A total of 528 doctors participated in the study. The result of the physical examination and additional tests, as well as the recommendations of scientific societies are the most important in deciding whether to start antibiotic therapy. Patient pressure (*p* < 0.011) and workload (*p* = 0.021) significantly influenced the decision to prescribe an antibiotic among primary care physicians and physicians in the course of specialization, who fear of legal consequences (*p* < 0.001). The habits of other physicians (*p* < 0.001) working at the same facility appeared to be additionally important. **Conclusions**: The decision to implement antibiotic therapy in upper respiratory tract infections is influenced by several factors that depend on the doctor (including place of work and seniority) and the patient (clinical symptoms, expectation of antibiotic prescription). The physician’s level of knowledge contributes to reducing antibiotic prescribing. Considering the factors associated with the level of knowledge and awareness, together with a high prevalence of self-medication with antibiotics in Polish population, there is a strong need to design educational interventions aimed at reducing inappropriate antibiotic prescribing and preventing antibiotic resistance in Poland.

## 1. Introduction

Antibiotics have been used to treat diseases caused by bacteria since ancient times. The Ebers papyrus, dating back to approximately 1550 BC, describes the treatment of bread with mold. Later discoveries in the early 20th century initiated the era of antibiotic therapy, which changed the face of modern medicine [1]. It is believed that the use of antibiotics has contributed to the extension of human life by an average of 23 years [2]. The first studies on the existence of substances that exhibit antimicrobial activity were observations in the late 19th century conducted by Paul Ehrlich on the effect of salvarsan in the treatment of patients infected with syphilis spirochete [3]. In contrast, the first natural antibiotic was penicillin, discovered by Alexander Fleming in 1928, which is still used today to treat diseases caused by group B streptococci [4]. In 1935, Gerhard Domagk demonstrated the efficacy of a red dye called prontosil against streptococcal infections, which contributed to the discovery of a whole group of sulfonamide antibiotics [5]. However, as early as the 1940s, the phenomenon of antibiotic resistance, for example, in the case of streptomycin’s effect on mycobacteria, was described [6]. Nowadays, more than 100 years after the discovery of antibiotics, there is increasing talk of growing antibiotic resistance, contributing to an estimated 1.27 million deaths worldwide due to multiresistant bacteria [7]. Antibiotic resistance is the inherited ability of a microorganism to produce a mechanism that allows it to counteract the effects of an antibiotic despite its high concentrations [8]. Human pathogens use two mechanisms to acquire antibiotic resistance. The first mechanism involves mutated resistance genes passed on through reproduction, and the second mechanism involves acquiring resistance genes from other microorganisms [9]. Increasing antibiotic resistance is a result of the overuse or misuse of antibiotics, and their presence in the environment due to agricultural activities and in wastewater as a result of improper disposal [10]. It is indicated that regarding healthcare, the main irregularities giving rise to the development of antibiotic resistance are the use of antibiotics in the treatment of infections caused by viruses, the use of too-low doses of antibiotics, and/or inappropriate drug selection. These errors are related to insufficient knowledge among doctors and the phenomenon of self-medication among patients [11].

Currently, new antimicrobial drugs that should be effective against more strains of bacteria with acquired resistance to existing antibiotics are being developed. However, this is a difficult, not always cost-effective, and, above all, long process. According to recent estimates, if appropriate measures to reduce antibiotic resistance are not implemented by 2050, 10 million people worldwide will die each year from previously easily treatable infections [12]. One such measure is the document prepared by the WHO in 2018 on the need to classify groups of antibiotics and use them adequately and rationally [13]. The key to protecting antibiotics is to use them appropriately, following the knowledge of the microorganisms causing the disease being treated, considering local bacterial resistance and the individual immune status of the patient, and knowing the pharmacokinetics and dynamics of the preparations in question [14].

Since in Poland, most antibacterial drugs are sold only by prescription, it is mainly up to physicians to decide whether to prescribe antibiotic treatment. Information on the amount of antibiotics used worldwide is published annually by the WHO. In addition, these data are included in an OECD report detailing the average amount of antibiotics used in Europe. National antimicrobial consumption is expressed as “Defined Daily Doses” (DDD) per 1000 individuals per day, which can be interpreted as the average number of people per 1000 individuals treated with antimicrobials each day [15]. A 2023 OECD report shows that Poland is the top country in terms of antibiotic prescriptions in the European Union. The average for all OECD countries is 13 DDD per 1000 individuals per day. In Europe, only physicians in Greece (22 DDD per 1000 individuals per day), Romania (24 DDD per 1000 individuals per day), and Bulgaria (22 DDD per 1000 individuals per day) prescribe more antibiotics than in our country. In contrast, the least antibiotics are prescribed by primary care physicians from Austria (7 DDD per 1000 individuals per day), Germany (8 DDD per 1000 individuals per day), and the Netherlands (8 DDD per 1000 individuals per day). A comparison of 2019 and 2021 data on the average amount of antibiotics prescribed in OECD countries shows a decrease in the amount of antibiotics prescribed in Europe (from 17 to 13 DDD per 1000 individuals per day). The report’s authors explain this phenomenon through changes in guidelines for prescribing antimicrobial treatment and a decrease in infectious diseases as a result of restrictions on preventing microbial transmission during the COVID-19 pandemic [16]. As for Poland, according to data from the National Health Fund, physicians working in primary healthcare are responsible for 80–90% of antibiotic prescriptions prescribed for the treatment of upper respiratory tract infections [17]. A study of a population of patients in long-term care facilities in Poland found that one of the reasons for the high number of antibiotic prescriptions was the use of empirical therapy without performing microbiological tests to confirm the bacterial etiology of the infection and the need for antibiotic treatment [18]. Belgian researchers conducted an observation of video recordings of doctors’ visits for infections in Night Care, which showed that one of the reasons why a doctor prescribed an antibiotic was pressure from the patient who presented their symptoms as severe [19]. Some doctors, as well as patients, see antibiotic therapy as a way to shorten the duration of an infection [20]. A survey conducted among medical students at the Warsaw Medical University in 2018 on the level of knowledge about antibiotics and antibiotic resistance, as well as a compilation of results from surveys conducted among Polish physicians participating in the Center for Medical Postgraduate Education (CMPE) course in 2019–2020, indicated sufficient knowledge about the indications for antibiotic therapy and the causes of antibiotic resistance [21,22]. However, similar studies targeting primary care physicians, who are the first line of contact for patients with infections, are lacking.

As members of the Polish Society of Family Medicine Scientific Section, we hypothesized that there are factors associated with physicians and patients that might influence the antibiotics prescription. Therefore, we aimed to identify these factors in the Polish primary healthcare setting and analyze them in relation to prescribing of antibiotics for upper respiratory tract infections by primary care physicians assessing their attitudes toward antibiotic resistance, and knowledge of the principles of antibiotic use.

For better clarity of the survey’s methodology, we are providing an English-language version of the survey as Supplementary Materials.

## 2. Results

### 2.1. Characteristics of the Study Group

A total of 528 doctors participated in the study, the vast majority of whom were women (78.6%). Among the respondents, most live in cities of >500 thousand residents (34.5%). Primary healthcare is the main place of work for 449 (85.0%) of the respondents. The average seniority of the study group is 10.7 years of work. A detailed description of the study group is presented in Table 1.

### 2.2. Parameters Affecting a Doctor’s Decision to Prescribe an Antibiotic

Using a 5-degree Likert scale, the criteria guiding the decision to include an antibiotic were evaluated.

It was shown that the most important criterion in deciding whether to include antibiotic therapy is the result of the physical examination (71% of respondents point it as the most important) and the recommendations of scientific societies (64% of respondents point it as the most important). The results of additional tests are also an important element.

The criterion with a low impact on decision-making is pressure from the patient, the workload on a given day, and the amount of time dedicated to the patient during the visit. In specific clinical situations, it has been shown that thick, green nasal discharge, persistent cough, and an acute sore throat given in the patient’s interview declaratively are not the most significant factors influencing the final decision.

It was shown that in physicians working mostly in primary healthcare, patient pressure (*p* < 0.011) and workload (*p* = 0.021) have a greater impact on the decision than in doctors from other groups. On the other hand, the comparison based on the career stage showed that physicians in the course of specialization are significantly more likely to be guided by fear of legal consequences (*p* < 0.001), the habits of other doctors working at the same facility (*p* < 0.001), pressure from the patient (*p* < 0.001), and workload (*p* = 0.048), when deciding whether to prescribe an antibiotic. A detailed summary of the results is shown in Table 2.

Analyzing the correlation between seniority and factors affecting the decision for antibiotic therapy showed a positive correlation with thick green nasal discharge (r = 0.423, *p* < 0.001), persistent cough (r = 0.151, r = 0.001), and acute sore throat (r = 0.150, *p*= −0.001). Interestingly, an inverse correlation was shown with workload, amount of time for the patient during the visit, patient pressure, or fear of legal consequences. A detailed summary of the correlations is shown in Table 3.

### 2.3. Daily Clinical Practice

For the question assessing daily clinical practice and situations in which a doctor would decide to implement an antibiotic, 465 (88.1%) respondents correctly indicated that they would start the treatment in case of symptoms lasting more than 14 days, worsening symptoms of unilateral nasal leakage, and fever above 38 °C. As many as 85 (16.1%) doctors would start antibiotic therapy for a cough lasting 3 weeks, and five (0.9%) would include it in response to a single tick bite in a non-endemic area.

### 2.4. Concerns About Side Effects of Antibiotic Therapy

In the study group, 330 physicians said they were concerned about the side effects of the antibiotic therapy they were prescribing. There were no differences between women and men (63.4% vs. 58.4%; *p* = 0.539), career stage (specialists vs. residents vs. others; 61.5% vs. 61.1% vs. 79.4%; *p* = 0.108), and main place of work (primary healthcare vs. others; 61.3% vs. 69.6%; *p* = 0.152). In their practice, physicians most often observe complications resulting from dysbiosis, gastrointestinal symptoms, and interactions with the patient’s daily medications.

### 2.5. Patients

In their daily practice, 95.6% of doctors declare that they encounter attempts to force antibiotic therapy for adult patients, and 83.7% of doctors observe this phenomenon when caring for children. The doctors’ declarations show that the most frequent reasons for the need for an antibiotic claimed by patients are the fact that they know their body and only an antibiotic can help them, and that they need to recover quickly. The frequency of refusal to implement antibiotic therapy despite the doctor’s recommendation was also assessed. The majority of doctors rarely encounter a situation where a patient refuses a recommended antibiotic for themselves or their child (66.3% and 65.1%, respectively).

### 2.6. Changes in the Number of Issued Antibiotic Prescriptions and Doctors’ Opinions on Antibiotic Resistance

When asked about changes in the number of antibiotic prescriptions for the treatment of upper respiratory tract infections, the majority of doctors (47.5%) said they were prescribing fewer antibiotics than 2 years ago. A similar number of doctors (44.1%) still prescribe the same amount of antibiotics, and only 8.4% admitted that they issue more prescriptions. Doctors who prescribe fewer antibiotics mainly cite the increase in the availability of 3-in-1 Combo tests (85.3%), Strep tests (72.1%), and rapid CRP tests (62.5%) in primary healthcare as reasons for the change, but also point to training (improving professional competence—68.9%), the growing awareness (51.4%), and several scientific reports on the antibiotic resistance phenomenon (46.6%).

### 2.7. Self-Medication

518 (98.1%) physicians say they encounter attempted self-medication with antibiotics by their patients in their daily practice. Doctors indicate that self-medication is most often attempted by women (76.6%), older people (44.1%), urban residents (32.4%), and people with primary education (31.1%).

### 2.8. Level of Knowledge

Five proprietary questions were used to assess knowledge. A total of 96.4% of doctors correctly indicate the first-line antibiotic in non-recurrent streptococcal pharyngitis without known hypersensitivity reaction. In contrast, only 41.5% of doctors know the recommended second-line antibiotic for recurrent streptococcal pharyngitis. Moreover, only 16.5% of doctors correctly indicated the indications for immediate antibiotic therapy in a child. A total of 73.9% of doctors correctly indicated the principles of treatment of acute bronchitis in children, and 44.7% correctly assessed the need for additional tests in a burdened adult patient. On average, doctors scored 2.73 points, four doctors scored 0 points, and 22 (4.2%) scored the maximum number of points. A detailed summary of questions and answers assessing knowledge of antibiotic therapy is presented in Table 4.

Comparing the results obtained from the knowledge test showed that physicians practicing mainly in primary healthcare obtained higher scores than their colleagues (2.77 vs. 2.51, *p* = 0.023). Interestingly, in the comparison of career stages, it was shown that those in the course of specialization obtained the highest scores, higher than those with specialization. This was also confirmed by correlation analysis, which showed a negative correlation between seniority and total scores (r = −0.160, *p* = 0.001) (Table 5).

On the other hand, the correlation analysis between the sum of the scores obtained in the knowledge test and the factors influencing the decision on antibiotic therapy showed an inverse correlation with reporting thick, green nasal discharge (−0.176, *p* < 0.001) and a positive correlation with using the recommendations of scientific societies (r = 0.110, *p* = 0.012) (Table 6).

We checked whether the participating physicians were aware of the existence of various sources of information on antibiotic treatment and antibiotic resistance. Doctors most often obtain their knowledge from scientific guidelines on antibiotic resistance in the treatment of upper respiratory tract infections (80.9%) and scientific conferences (47.9%). It turned out that the largest number of doctors (33.7%) know at least two sources from which to obtain knowledge on these topics, and these are mainly scientific guidelines on the topic of antibiotic resistance in the treatment of upper respiratory tract infections and scientific conferences on this topic. Unfortunately, there is also a group of physicians (6.6%) who do not know any way to obtain information on antibiotic use.

### 2.9. Familiarity with Campaigns vs. Mean Score Obtained in the Knowledge Test

It was shown that those who knew at least one of the campaigns on rational antibiotic therapy obtained significantly higher scores on the knowledge test (2.76 ± 1.0 vs. 2.41 ± 1.0; *p* = 0.034). A detailed comparison is shown in Table 7. There was no correlation between the number of known campaigns on rational antibiotic therapy and the total score obtained in the knowledge test (r = 0.004, *p* = −0.991).

## 3. Discussion

The purpose of this study was to evaluate factors influencing the prescribing of antibiotics in upper respiratory tract infections by primary care physicians in Poland, attitudes toward antibiotic resistance, and knowledge of the principles of antibiotic use. Our results show that factors such as the result of the physical examination, the results of additional tests, and the recommendations of scientific societies are the most important in deciding whether to start antibiotic therapy. Importantly, it turned out that non-meritorious considerations were also important. Patient pressure and workload significantly influenced the decision to prescribe an antibiotic among primary care physicians and physicians in the course of specialization, for whom fear of legal consequences and the habits of other physicians working at the same facility appeared to be additionally important. Interestingly, as a doctor’s seniority and experience increase, factors such as workload, amount of time for the patient during the visit, patient pressure, or fear of legal consequences lose their importance in the decision to prescribe antibiotic therapy.

A study in Denmark analyzed data showing the amount of antibiotic prescribing by physicians working in Night and Holiday Care between 2014 and 2017. The researchers created the concept of “antibiotic prescribing tendency” (APT), which reflects the likelihood of an individual family doctor prescribing an antibiotic compared to the average family doctor. An APT > 1 means that a family doctor is more likely to prescribe antibiotics than the average family doctor, and an APT < 1 means that a family doctor is less likely to prescribe antibiotics than the average family doctor. The obtained results coincided with ours. The authors of a Danish study showed that a high APT is associated with a high number of medical visits (workload), a doctor’s young age, and less seniority in Night Care [23].

In 2023, a review of papers on inappropriate antibiotic prescribing by primary care physicians in developed European countries was published, where the family doctor acts as a “gatekeeper” in the healthcare system. Seventeen studies from 2021 and before were included in the analysis, showing the existence of as many as 45 factors determining inappropriate antibiotic prescribing. These factors were divided into physician-dependent and patient-dependent. It was indicated that on the physician’s part, antibiotic therapy was most often inappropriately recommended for patients with comorbidities, so patients with an increased risk of complications of infectious disease. Such a situation is associated with fear of negative legal consequences of not prescribing the treatment, which was also an important argument for including antibiotic therapy in our study. In contrast to our results, it was noted that doctors with seniority of more than 10 years are more likely to prescribe antibiotics, paying attention to the doctor–patient relationship and assuming that the patient expects a prescription. Similarly to our observations, familiarity with clinical practice guidelines had a positive effect on the number of antibiotics prescribed. Knowledge about diseases that require the use of antibiotics, their symptoms, and the type of drug needed to use is provided to future doctors studying in Poland, but taking into account the growing antibiotic resistance and emerging new methods of treatment, current guidelines can be found in documents, such as “Recommendations for management of community-acquired respiratory infections (2016)” available on the website of the National Antibiotic Protection Program for all doctors practicing in Poland (www.antybiotyki.edu.pl, accessed on 16 February 2025). However, not all physicians are equally interested in self-education. 

As for factors on the patient’s part, it was pointed out that antibiotics were more often prescribed to those who are sicker for more than 7 days, those with acute symptoms of infection (especially fever), and comorbidities. Another interesting phenomenon is the more frequent prescription of an antibiotic to a patient who had already been treated with one in the previous year, or to people who had received antibiotic treatment deemed effective for symptoms similar to the current ones. It is also noteworthy that the patient’s request for an antibiotic prescription is one of the main reasons for inappropriate treatment, which is consistent with our results [24].

German researchers noted that about 40–45% of antibiotic prescriptions are issued within the primary healthcare setting. They analyzed patient records from visits to family physicians (adult patients) and pediatricians for the frequency of antibiotic prescriptions in conjunction with the diagnosis. As many as 31.2% of adult patients with an infection received an antibiotic from their family doctor, while only 9.1% of children were treated with antibiotics by pediatricians. Even despite the diagnosis of a viral infection of an undetermined origin, physicians prescribed an antibiotic (1.5% of family physicians and 6.4% of pediatricians). Both in the adult and pediatric populations, older patient age was associated with the decision to prescribe an antibiotic. Adults with comorbidities and children with asthma were more likely to be treated with antibiotics than the generally healthy population. In the present study, patient age was not associated with the decision to start antibiotic therapy [25].

As mentioned earlier, most antibiotics are prescribed in primary healthcare for respiratory infections, followed by urinary tract infections [26,27]. Even though most upper respiratory tract infections are caused by viruses, antibiotics are still prescribed for conditions such as colds, acute rhinitis, or acute bronchitis. For upper respiratory tract infections, symptoms such as fever, purulent sputum, plaque on tonsils, and abnormal test results can influence the decision to prescribe antibiotics. However, it is estimated that young doctors’ knowledge of treating upper respiratory tract infections is limited. Although the etiology of these infections is overwhelmingly viral, the inclusion of antibiotic therapy with the expectation of effective treatment is observed. This is one of the factors generating increasingly widespread antibiotic resistance [28].

One of the key goals of the World Health Organization (WHO) is rational antibiotic prescription (RAP) as an effective strategy to reduce morbidity and mortality from infection. Implementation of a RAP program consisting of bi-weekly educational seminars, auditing physicians’ antibiotic prescriptions, and providing feedback to physicians has been shown to reduce irrational antibiotic prescribing (IRAP) from 43 to 20.6%, and the mortality rate from 10.4 to 8% [29,30]. In 2015 WHO adopted the Global Action Plan on Antimicrobial Resistance. More than 120 countries have developed a national action plan, but many places are failing to implement antimicrobial resistance (AMR) policies. In recent years, countries have tried to formulate AMR policies based on evidence, data, and scientific consensus on AMR transmission pathways. However, advocacy also relies heavily on economic, political, and social correlations between health systems, food production, trade, and national productivity costs [31].

The results of our study showed that in addition to the availability of diagnostic tests in primary healthcare, the most important factor influencing the reduction in antibiotic prescriptions is the ability to improve professional competence through training and scientific reports on antibiotic resistance. Moreover, recommendations from scientific societies significantly influenced the decision to include antibiotics and were the main source of knowledge for more than 80% of doctors. Scientific conferences were indicated in second place. We also observed a significant correlation between a higher score in the knowledge test and familiarity with at least one campaign on antibiotic resistance. Undoubtedly, educational activities aimed at physicians, including those working in primary healthcare, are an important strategy for its prevention. Sweden’s Strategic Program for Combating Antibiotic Resistance (Strama) identified the best conditions for primary healthcare that can enable physicians to reflect on their prescribing of antibiotics, help reduce uncertainty about appropriate treatment of acute respiratory infections, educate primary care physicians about appropriate prescribing, and facilitate more patient-centered care. These conditions include knowledge, reflective collegial dialogs, a well-organized workplace, and a collaborative team [32]. An Italian study found that healthcare workers, including physicians, differ in their knowledge, attitudes, and behaviors regarding antibiotic use. The spread of antibiotic resistance was higher in regions where awareness and knowledge of antibiotic use were lower [33]. Portugal also highlighted the important aspect of educating physicians to reduce antibiotic resistance. The survey was designed as a tool to assess the attitudes and knowledge underlying antibiotic prescribing by physicians, with the aim of understanding and improving antibiotic use in both hospital and primary healthcare settings. Physicians’ attitudes and knowledge about antibiotic prescribing and resistance were assessed among 61 primary care physicians and 50 hospital physicians. This study emphasized that the survey identifies factors influencing antibiotic prescribing while serving as a basis for developing effective educational interventions [34]. Researchers in the UK did not just stop at the stage of verifying physicians’ knowledge, but created an educational tool. They relied on the premise that educating prescribers contributes to optimizing antimicrobial use, but at the same time, there is a need to identify key educational goals in this regard. Using qualitative methods, they verified the knowledge, attitudes, and behaviors of young doctors regarding antimicrobial prescribing. In doing so, they developed an educational tool that provided knowledge on the principles of antimicrobial prescribing, diagnosis of infections, clinical review of patients with infections, prescribing in the context of antimicrobial resistance, and the role of laboratory tests and test results in prescribing [35]. In Spain, a country with one of the highest antibiotics uses in Europe, attention has been paid to educating physicians from the earliest stages of their careers. A qualitative study found that young physicians were guided by the opinions of senior colleagues and their workload in deciding whether to start antibiotic therapy. Appropriate educational interventions have the potential to improve inappropriate work habits [36].

The authors are aware of the limitations of the above study, which is undoubtedly the methodology of data collection using an online survey. However, it should be noted that CAWI-type surveys are currently a common research method to reach a wide audience. In addition, the lack of representativeness of our group to physicians working in primary care is also a limitation.

## 4. Materials and Methods

We conducted a CAWI (Computer-Assisted Web Interview) survey, carried out using a proprietary survey distributed online from May to July 2024. The anonymous survey was distributed via the social network Facebook.com and the Instagram application in groups (with closed access) associating doctors working in primary healthcare in Poland. In addition, information about the survey was disseminated by email using the Polish Society of Family Medicine’s mailing list directed to physicians. The survey was aimed at people over the age of 18, with a license to practice medicine, and working in primary healthcare. Before participating in the survey, the participant was presented with the objectives of the study and its methodology, after which they gave informed consent to participate in the study. At each stage of the survey, the respondent had the opportunity to discontinue participation without giving a reason.

The proprietary survey, based on a literature review with consideration of other surveys on antibiotic resistance aimed at physicians, consisted of 26 questions, 25 of which were closed questions. Four questions were multiple choice, and six questions required the evaluation on a 5-degree Likert scale.

The survey was divided into six sections. The first section included questions on sociodemographic variables such as age, gender, and place of residence. In addition, respondents were asked about their type of specialty, main place of work, and seniority. The next section included questions about doctors’ daily practice regarding factors influencing the decision to prescribe antibiotics, the choice of additional tests, and patient symptoms influencing the treatment decision. In the next section, we asked respondents about their attitude towards antibiotic treatment. Respondents answered questions about factors influencing their decisions to prescribe antibiotics and about concerns regarding side effects and their type. They were then asked about patients’ attitudes toward antibiotic treatment, whether patients put pressure on doctors to prescribe an antibiotic, and what reasons they gave for requesting such treatment. A question was also asked about whether respondents had encountered self-medication among their patients or patients refusing to take an antibiotic despite recommendations. The section on antibiotic knowledge included 5 questions on the correct choice of antibiotic type in pharyngitis, indications for including antibiotic treatment in otitis media, how to treat bronchitis in a child, and recommendations for additional tests in a patient with asthma and upper respiratory tract infection symptoms. In the last section, we tested awareness of antibiotic resistance among physicians by asking whether respondents were familiar with campaigns on the need to reduce antibiotic prescriptions, how their antibiotic prescriptions for upper respiratory tract infections have changed, and the reasons for the reduction in prescriptions.

The English-language version of the survey has been included as Supplementary Materials.

### Statistical Analysis

The variables analyzed were qualitative and quantitative. The Shapiro–Wilk Test was used to assess the normality of distribution. Comparisons between quantitative variables were made using non-parametric Mann–Whitney U tests and the Kruskal–Wallis Test. Spearman’s correlation test was used to assess the degree of correlation between quantitative variables. Comparisons between qualitative variables were made using the Chi-square test.

The significance level was established at 0.05. The analyses were carried out using Statistica 13.0 by StatSoft Inc. (Cracow, Poland).

## 5. Conclusions

The decision to implement antibiotic therapy in upper respiratory tract infections is influenced by several factors that depend on the doctor (including place of work and seniority) and the patient (clinical symptoms, expectation of antibiotic prescription). The physician’s level of knowledge contributes to reducing antibiotic prescribing. The main sources of education are guidelines from scientific societies and scientific conferences. Considering the factors associated with level of knowledge and awareness, together with a high prevalence of self-medication with antibiotics in Polish population, there is a strong need to design educational interventions aimed at reducing inappropriate antibiotic prescribing and preventing antibiotic resistance in Poland [37].

## Figures and Tables

**Table 1:** Characteristics of the study group.

Variable		N (%)/M ± SD
Sex	Female	415 (78.6)
	Male	113 (21.4)
Place of residence	Countryside	71 (13.4)
	City of less than 100 thousand residents	130 (24.6)
	City of 100–500 thousand residents	145 (27.5)
	City of >500 thousand residents	182 (34.5)
Professional career stage	Specialists	291 (55.1)
	During specialization	203 (38.4)
	Other	34 (6.5)
Main place of work	Primary healthcare	449 (85.0)
	Other	79 (15.0)
Job seniority [age]		10.73 ± 9.3

N—number, M—Mean, SD—Standard deviation.

**Table 2:** Factors influencing antibiotic use decisions for the entire group and with a breakdown based on main workplace and career stage.

Variable	The Whole Group M ± SD	Main Place of Work			Professional Career Stage			
		Primary Care Physician M ± SD	Other M ± SD	*p*	Specialist M ± SD	During Specialization M ± SD	Other	*p*
Patient’s age	3.65 ± 1.1	3.65 ± 1.1	3.65 ± 1.2	0.824 #	3.67 ± 1.2	3.67 ± 1.0	3.41 ± 1.0	0.289 *
Duration of the infection	4.04 ± 0.9	4.02 ± 0.9	4.15 ± 0.9	0.297 #	4.04 ± 0.9	4.06 ± 0.9	3.91 ± 0.8	0.198 *
Thick, green nasal discharge	1.67 ± 1.0	1.65 ± 0.9	1.73 ± 1.0	0.704 #	1.85 ± 1.1	1.39 ± 0.7	1.65 ± 1.1	<0.001 *
Persistent cough	2.63 ± 1.1	2.59 ± 1.1	2.87 ± 1.0	0.021 #	2.73 ± 1.1	2.51 ± 1.0	2.48 ± 1.1	0.057 *
Acute sore throat	2.56 ± 1.1	2.52 ± 1.1	2.72 ± 1.2	0.197 #	2.64 ± 1.2	2.48 ± 1.1	2.29 ± 1.2	0.082 *
The result of the physical examination	4.66 ± 0.6	4.65 ± 0.6	4.72 ± 0.5	0.478 #	4.67 ± 0.6	4.66 ± 0.6	4.62 ± 0.6	0.999 *
The result of additional tests (for example CRP, strep test)	4.33 ± 0.7	4.31 ± 0.7	4.46 ± 0.7	0.065 #	4.32 ± 0.8	4.37 ± 0.7	4.24 ± 0.7	0.613 *
Comorbidities	4.00 ± 0.9	3.96 ± 0.9	4.2 ± 0.9	0.024 #	4.02 ± 0.9	3.97 ± 0.9	3.91 ± 0.8	0.198 *
Recommendations of scientific societies	4.56 ± 0.6	4.53 ± 0.7	4.70 ± 0.6	0.018 #	4.57 ± 0.6	4.57 ± 0.7	4.41 ± 0.7	0.989 *
Workload	1.99 ± 0.1	2.05 ± 1.0	1.65 ± 0.8	0.005 #	1.86 ± 1.0	2.14 ± 1.1	2.18 ± 1.1	0.032 *
Availability of additional tests	3.75 ± 1.1	3.76 ± 1.1	3.72 ± 1.0	0.556 #	3.62 ± 1.2	3.89 ± 1.0	4.18 ± 0.9	0.048 *
Amount of time for the patient during the visit	2.50 ± 1.3	2.54 ± 1.3	2.25 ± 1.1	0.101 #	2.34 ± 1.2	2.67 ± 1.3	2.74 ± 1.3	0.111 *
Belief in the value of additional tests	4.24 ± 0.9	4.25 ± 0.9	4.17 ± 0.9	0.369 #	4.2 ± 0.9	4.28 ± 0.8	4.12 ± 0.9	0.591 *
Habits of other doctors working at the same facility	1.41 ± 0.7	1.39 ± 0.7	1.52 ± 0.8	0.085 #	1.24 ± 0.6	1.65 ± 0.9	1.47 ± 0.7	<0.001 *
Fear of legal consequences if health deteriorates	2.26 ± 1.0	2.28 ± 1.1	2.16 ± 1.0	0.387 #	2.04 ± 0.9	2.52 ± 1.1	2.59 ± 1.1	<0.001 *
Pressure from the patient	2.05 ± 0.9	2.10 ± 1.0	1.78 ± 0.7	0.011 #	1.86 ± 0.8	2.33 ± 1.0	2.01 ± 0.8	<0.001 *

# Mann–Whitney U Test; * Kruskal–Wallis Test.

**Table 3:** Correlation between seniority and factors influencing the decision to implement antibiotic therapy.

Variable	Seniority	
	r	*p*
Patient’s age	0.052	0.230
Duration of the infection	0.014	0.754
Thick, green nasal discharge	0.423	<0.001
Persistent cough	0.151	0.001
Acute sore throat	0.150	0.001
The result of the physical examination	−0.030	0.485
The result of additional tests (for example CRP, strep test)	−0.022	0.616
Comorbidities	0.122	0.005
Recommendations of scientific societies	0.010	0.811
Workload	−0.170	<0.001
Availability of additional tests	−0.177	<0.001
Amount of time for the patient during the visit	−0.097	0.026
Belief in the value of additional tests	0.002	0.960
Habits of other doctors working at the same facility	−0.184	<0.001
Fear of legal consequences if the patient’s condition deteriorates	−0.217	<0.001
Pressure from the patient	−0.171	<0.001

**Table 4:** Summary of questions assessing knowledge of treatment of respiratory infections.

Question		N (%)
What is the first-line antibiotic for non-recurrent streptococcal pharyngitis in a patient with no known hypersensitivity reaction?	**Phenoxymethylpenicillin**	509 (96.4)
	Amoxicillin with clavulanic acid	7 (1.3)
	Clindamycin	3 (0.6)
	Cefuroxime axetil	5 (0.9)
	None of the above	4 (0.8)
What is the second-line antibiotic for recurrent symptoms of streptococcal pharyngitis or failure of first-line treatment without an identified cause of recurrence?	**Clindamycin**	219 (41.5)
	Cefuroxime axetil	126 (23.9)
	Amoxicillin	68 (12.9)
	Levofloxacin	4 (0.7)
	Azithromycin	37 (7.0)
	None of the above	74 (14.0)
In what situation would you decide to start antibiotic therapy in a child diagnosed with otitis media? [multiple choice question]	Immediately upon diagnosis of unilateral acute otitis media in a child under 2 years of age	182 (34.5)
	Immediately in a child with a high fever above 39 degrees C and severe pain regardless of age	241 (45.6)
	In case of no improvement after 24 h of anti-inflammatory treatment	79 (15.0)
	In case of no improvement after 48–72 h of symptoms in a child younger than 6 months	201 (38.1)
	None of the above	77 (14.6)
	**Correct answer [option 2 only]**	87 (16.5)
What treatment would you administer to a child presenting to the primary care physician’s office with acute bronchitis on the third day of symptoms, in good general condition, without respiratory effort or drops in saturation? [multiple choice question]	Inhaled corticosteroids	54 (10.2)
	Amoxicillin with clavulanic acid	3 (0.6)
	Levofloxacin	1 (0.2)
	Inhalations with 3% salt	90 (17.0)
	Hydration, cleaning the nose, antipyretics, possible administration of a mucolytic or peripheral antitussive cough medicine depending on the type of cough	506 (95.8)
	None of the above	6 (1.1)
	**Correct answer [option 4 only]**	390 (73.9)
Which additional test would you order for a patient with bronchial asthma presenting to your primary care physician’s office with symptoms lasting for 2 days: runny nose, productive cough, fever over 38 °C, headache? [multiple choice question]	Rapid CRP test	160 (30.3)
	Strep test	11 (2.1)
	CBC and CRP from venous blood	75 (14.2)
	So-called Combo antigen test (influenza, COVID, RSV)	422 (80.0)
	Nasal swab culture	1 (0.2)
	I wouldn’t use any additional tests	74 (14.1)
	**Correct answer [option 3 only]**	236 (44.7)
Total points from the knowledge test	0	4 (0.8)
	1	61 (11.6)
	2	155 (29.4)
	3	184 (34.8)
	4	102 (19.3)
	5	22 (4.2)
	M ± SD	2.73 ± 1.1

N—number, M—Mean, SD—Standard deviation.

**Table 5:** Comparison of the level of knowledge by workplace and career stage.

	The Whole Group M ± SD	Main Place of Work			Professional Career Stage			
		Primary Care Physician M ± SD	Other M ± SD	*p*	Specialist M ± SD	During Specialization M ± SD	Other	*p*
Level of knowledge (M ± SD)	2.73 ± 1.1	2.77 ± 1.1	2.51 ± 1.1	0.023 #	2.62 ± 1.0	2.92 ± 1.1	2.55 ± 1.1	0.015 *

M—Mean, SD—Standard deviation, # Mann–Whitney U Test; * Kruskal–Wallis Test.

**Table 6:** Correlation between the level of knowledge and factors influencing the decision to start antibiotic therapy.

Variable	Level of Knowledge	
	r	*p*
Patient’s age	−0.021	0.630
Duration of the infection	−0.0523	0.230
Thick, green nasal discharge	−0.176	<0.001
Persistent cough	−0.071	0.105
Acute sore throat	−0.076	0.071
The result of the physical examination	0.043	0.329
The result of additional tests (for example CRP, strep test)	−0.028	0.522
Comorbidities	−0.016	0.971
Recommendations of scientific societies	0.110	0.012
Workload	−0.039	0.362
Availability of additional tests	0.089	0.840
Amount of time for the patient during the visit	−0.056	0.198
Belief in the value of additional tests	−0.043	0.921
Habits of other doctors working at the same facility	−0.037	0.401
Fear of legal consequences if health deteriorates	−0.028	0.517
Pressure from the patient	−0.054	0.215

**Table 7:** Comparison of mean scores obtained in the knowledge test about knowledge of individual educational campaigns on rational antibiotic therapy.

Campaign		M ± SD	*p*
The Supreme Medical Board’s campaign “Education on the rational use of antibiotics in upper respiratory tract diseases”.	Yes	2.38 ± 1.1	0.011
	No	2.78 ± 1.0	
The article “Rational antibiotic therapy in upper respiratory tract infections—recommendations versus patient perspective. Analysis of the results of the IPSOS 2022 survey”.	Yes	2.68 ± 1.1	0.503
	No	2.76 ± 1.1	
Educational campaign with the “9 out of 10 throat infections are caused by viruses” slogan.	Yes	2.71 ± 0.9	0.911
	No	2.73 ± 1.1	
Scientific conferences on the topic of antibiotic resistance in the treatment of upper respiratory tract infections.	Yes	2.74 ± 1.0	0.885
	No	2.72 ± 1.1	
Scientific guidelines on the topic of antibiotic resistance in the treatment of upper respiratory tract infections.	Yes	2.75 ± 1.1	0.431
	No	2.63 ± 1.0	
Other than the listed activities with the theme of antibiotic resistance in the treatment of upper respiratory tract infections.	Yes	2.93 ± 1.0	0.016
	No	2.68 ± 1.0	
I am not aware of any activities on antibiotic resistance in the treatment of upper respiratory infections.	Yes	2.41 ± 1.0	0.034

## Data Availability

The original contributions presented in this study are included in the article/Supplementary Materials. Further inquiries can be directed to the corresponding author.

## Supplementary Materials

The following supporting information can be downloaded at: https://www.mdpi.com/article/10.3390/antibiotics14020212/s1. English version of the questionnaire used.

## References

[1] Elsayad K. (2023). What ancient Egyptian medicine can teach us. JCO Glob. Oncol..

[2] Hutchings M.I., Truman A.W., Wilkinson B. (2019). Antibiotics: Past, present and future. Curr. Opin. Microbiol..

[3] Vernon G. (2019). Syphilis and salvarsan. Br. J. Gen. Pract. J. R. Coll. Gen. Pract..

[4] Barrett M.P. (2023). The legacy of penicillin’s first patient. Pharmacol. Res. Off. J. Ital. Pharmacol. Soc..

[5] Christensen S.B. (2021). Drugs that changed society: History and current status of the early antibiotics: Salvarsan, sulfonamides, and β-lactams. Molecules.

[6] Crofton J., Mitchison D.A. (1948). Streptomycin resistance in pulmonary tuberculosis. Br. Med. J..

[7] Murray C.J., Ikuta K.S., Sharara F., Swetschinski L., Aguilar G.R., Gray A., Han C., Bisignano C., Rao P., Wool E. (2022). Global burden of bacterial antimicrobial resistance in 2019: A systematic analysis. Lancet.

[8] Huemer M., Mairpady Shambat S., Brugger S.D., Zinkernagel A.S. (2022). Antibiotic resistance and persistence-Implications for human health and treatment perspectives. EMBO Rep..

[9] Eisenreich W., Rudel T., Heesemann J., Goebel W. (2022). Link between antibiotic persistence and antibiotic resistance in bacterial pathogens. Front. Cell. Infect. Microbiol..

[10] Nava A.R., Daneshian L., Sarma H. (2022). Antibiotic resistant genes in the environment-exploring surveillance methods and sustainable remediation strategies of antibiotics and ARGs. Environ. Res..

[11] Uddin T.M., Chakraborty A.J., Khusro A., Zidan B.R.M., Mitra S., Emran T.B., Dhama K., Ripon M.K.H., Gajdács M., Sahibzada M.U.K. (2021). Antibiotic resistance in microbes: History, mechanisms, therapeutic strategies and future prospects. J. Infect. Public Health.

[12] Cook M.A., Wright G.D. (2022). The past, present, and future of antibiotics. Sci. Transl. Med..

[13] Sharland M., Pulcini C., Harbarth S., Zeng M., Gandra S., Mathur S., Magrini N., 21st WHO Expert Committee on Selection and Use of Essential Medicines (2018). Classifying antibiotics in the WHO Essential Medicines List for optimal use-be AWaRe. Lancet Infect. Dis..

[14] Moser C., Lerche C.J., Thomsen K., Hartvig T., Schierbeck J., Jensen P.Ø., Ciofu O., Høiby N. (2019). Antibiotic therapy as personalized medicine—General considerations and complicating factors. APMIS Acta Pathol. Microbiol. Et Immunol. Scand..

[15] World Health Organization Antibacterial Consumption: Total Consumption of Antibiotics Expressed as DDD per 1000 Inhabitants per Day. https://www.who.int/data/gho/data/indicators/indicator-details/GHO/antibacterial-consumption--total-consumption-of-antibacterials-expressed-as-ddd-per-1000-inhabitants-per-day.

[16] OECD (2023). Health at a Glance 2023: OECD Indicators.

[17] National Health Fund The Problem of Antibiotic Resistance. https://www.nfz.gov.pl/antybiotyki-stosuj-z-rozwaga/dla-lekarzy/.

[18] Wojkowska-Mach J., Brudło M., Topolski M., Bochenek T., Jachowicz E., Siewierska M., Różańska A. (2021). Antibiotic consumption in long-term care facilities in Poland and other European countries in 2017. Antimicrob. Resist. Infect. Control..

[19] Colliers A., Coenen S., Bombeke K., Remmen R., Philips H., Anthierens S. (2020). Understanding general practitioners’ antibiotic prescribing decisions in out-of-hours primary care: A video-elicitation interview study. Antibiotics.

[20] De Vocht K., Debie T., Bastiaens H., Anthierens S. (2021). The use of paracetamol for first-line treatment of acute sore throat. A descriptive generic qualitative study of GPs and patients. Eur. J. Gen. Pract..

[21] Sobierajski T., Mazińska B., Wanke-Rytt M., Hryniewicz W. (2021). Knowledge-based attitudes of medical students in antibiotic therapy and antibiotic resistance. A cross-sectional study. Int. J. Environ. Res. Public Health.

[22] Zgliczyński W.S., Bartosiński J., Rostkowska O.M. (2022). Knowledge and practice of antibiotic management and prudent prescribing among Polish medical doctors. Int. J. Environ. Res. Public Health.

[23] Huibers L., Vestergaard C.H., Keizer E., Bech B.H., Bro F., Christensen M.B. (2022). Variation of GP antibiotic prescribing tendency for contacts with out-of-hours primary care in Denmark—A cross-sectional register-based study. Scand. J. Prim. Health Care.

[24] Sijbom M., Büchner F.L., Saadah N.H., Numans M.E., de Boer M.G.J. (2023). Determinants of inappropriate antibiotic prescription in primary care in developed countries with general practitioners as gatekeepers: A systematic review and construction of a framework. BMJ Open.

[25] Sydenham R.V., Jarbøl D.E., Hansen M.P., Justesen U.S., Watson V., Pedersen L.B. (2022). Prescribing antibiotics: Factors driving decision-making in general practice. A discrete choice experiment. Soc. Sci. Med..

[26] Akkerman A.E., Kuyvenhoven M.M., Verheij T.J.M., van Dijk L. (2008). Antibiotics in Dutch general practice: Nationwide electronic GP database and national reimbursement rates. Pharmacoepidemiol. Drug Saf..

[27] Hawker J.I., Smith S., Smith G.E., Morbey R., Johnson A.P., Fleming D.M., Shallcross L., Hayward A.C. (2014). Trends in antibiotic prescribing in primary care for clinical syndromes subject to national recommendations to reduce antibiotic resistance, UK 1995-2011: Analysis of a large database of primary care consultations. J. Antimicrob. Chemother..

[28] Sánchez X., Landázuri A., Londo P., Manzano A., Moreno Roca A., Jimbo R. (2020). Knowledge, attitudes and practices in antibiotic use in family medicine students. J. Prim. Care Community Health.

[29] Murni I.K., Duke T., Kinney S., Daley A.J., Soenarto Y. (2015). Reducing hospital-acquired infections and improving the rational use of antibiotics in a developing country: An effectiveness study. Arch. Dis. Child..

[30] Haddadin R.N., Alsous M., Wazaify M., Tahaineh L. (2019). Evaluation of antibiotic dispensing practice in community pharmacies in Jordan: A cross sectional study. PLoS ONE.

[31] Chan O.S.K., Lam W.W.T., Fukuda K., Tun H.M., Ohmagari N., Littmann J., Zhou X.D., Xiao Y., Liu P., Wernli D. (2022). Antimicrobial resistance policy protagonists and processes-A qualitative study of policy advocacy and implementation. Antibiotics.

[32] Sundvall P.-D., Skoglund I., Hess-Wargbaner M., Åhrén C. (2020). Rational antibiotic prescribing in primary care: Qualitative study of opportunities and obstacles. BJGP Open.

[33] Barchitta M., Sabbatucci M., Furiozzi F., Iannazzo S., Maugeri A., Maraglino F., Prato R., Agodi A., Pantosti A. (2021). Knowledge, attitudes and behaviors on antibiotic use and resistance among healthcare workers in Italy, 2019: Investigation by a clustering method. Antimicrob. Resist. Infect. Control.

[34] Teixeira Rodrigues A., Ferreira M., Roque F., Falcão A., Ramalheira E., Figueiras A., Herdeiro M.T. (2016). Physicians’ attitudes and knowledge concerning antibiotic prescription and resistance: Questionnaire development and reliability. BMC Infect. Dis..

[35] Gharbi M., Moore L.S.P., Castro-Sánchez E., Spanoudaki E., Grady C., Holmes A.H., Drumright L.N. (2016). A needs assessment study for optimising prescribing practice in secondary care junior doctors: The Antibiotic Prescribing Education among Doctors (APED). BMC Infect. Dis..

[36] Molina-Romera G., Vazquez-Cancela O., Vazquez-Lago J.M., Montes-Villalba R.A., Roque F., Herdeiro M.T., Figueiras A. (2023). Knowledge, attitudes and practice regarding antibiotic prescription by medical interns: A qualitative study in Spain. Antibiotics.

[37] Kłoda K., Babicki M., Biesiada A., Gałązka-Sobotka M., Kowalska-Bobko I., Mastalerz-Migas A. (2024). Self-medication of adults and children in Poland—Results from outpatient health care physicians online questionnaire. Front. Pharmacol..

