# Supplementary material for: Factors Influencing Antibiotic Prescribing and Antibiotic Resistance Awareness Among Primary Care Physicians in Poland
Source: Antibiotics (Basel). 2025 Feb 19;14(2):212. doi: 10.3390/antibiotics14020212 (PMC11852036; doi:10.3390/antibiotics14020212)
Supplement: Supplementary file 1 [file antibiotics-14-00212-s001.zip › antibiotics-3338010-supplementary.pdf]

Survey on antibiotic use

Good morning,

We are members of the Polish Society of Family Medicine Scientific Section. We would like to invite you to participate in a survey aimed at finding out your opinion and experience on antibiotic prescribing and the phenomenon of antibiotic resistance.

The survey is fully anonymous and voluntary, please answer each question. You may opt-out at any stage of the survey.

1. Do you give your informed consent to participate in the survey?
   - Yes
   - No

2. Sex
   - Female
   - Male
   - Other than those listed
   - I don’t want to specify

3. Place of residence:
   - city of more than 500 thousand residents
   - city of 100-500 thousand residents
   - city of less than 100 thousand residents
   - countryside

4. In which specialty do you work?
   - family medicine doctor
   - specialist in pediatrics
   - specialist in internal medicine
   - doctor in the course of specialization in family medicine
   - doctor in the course of specialization in pediatrics
   - doctor in the course of specialization in internal medicine
   - other than those listed

5. How many years have you been practicing medicine for?

6. Main place of work:
   - primary healthcare
   - Night Care
   - hospital
   - private practice
   - outpatient specialty care

Daily practice section

1. To what extent from 1 to 5 do you follow the listed parameters when deciding whether to include an antibiotic in the treatment of a patient with an upper respiratory tract infection?
   1 - not at all 2 - rarely 3 - sometimes 4 - often 5 - always
   - age of the patient
   - duration of infection
   - thick, green nasal discharge reported by the patient
   - persistent cough reported by the patient
   - acute sore throat reported by the patient
   - the result of the physical examination
   - results of additional tests (e.g., Strep test, CRP, so-called Combo 3-in-1 antigen test)
   - information about the patient's comorbidities
   - recommendations of scientific societies

2. *(multiple choice question) A 40-year-old patient presented at his primary care physician's office with the following symptoms: fever for 2 days up to 38.2˚C, sore throat, runny nose, muscle pain. Physical examination showed enlarged cervical lymph nodes and swollen tonsils with plaques. What management(s) would you implement for the above patient?
   - prescribing antibiotic therapy without performing additional tests
   - CBC and CRP test
   - rapid CRP test
   - Strep test
   - throat swab culture
   - so-called Combo 3-in-1 antigen test (COVID, influenza, RSV)
   - prescribing symptomatic treatment
   - management other than the above

3. *(multiple choice question) In what situations would you decide to implement antibiotic therapy in your patient?
   - fever above 39˚C regardless of the duration of the illness
   - dry cough lasting more than 3 weeks
   - feeling of weakness reported by the patient
   - condition after a tick bite removed within 24 hours (bite in a non-endemic area)
   - deteriorating symptoms of unilateral nasal discharge lasting more than 14 days, with a fever above 38˚C
   - acute sore throat
   - in none of the above

Attitude section

1. On a scale of 1 to 5, to what extent do the following factors influence your decision to implement antibiotic therapy for upper respiratory tract infections?
   1 - no influence 2 - small influence 3 - moderate influence
   4 - strong influence 5 - very strong influence
   - workload
   - availability of additional diagnostic tests
   - amount of time for the patient during the visit
   - belief in the value of additional tests performed (e.g., CRP determination by rapid test, Combo test)
   - habits of other doctors working at the same facility
   - fear of legal consequences if the patient's condition deteriorates
   - pressure from the patient

2. When implementing antibiotic therapy in a patient with an upper respiratory tract infection, are you concerned about the occurrence of side effects of the treatment?
   - Yes
   - No

3. [If "Yes" in question 2] On a scale of 1 to 5, to what extent are you concerned about the following side effects and complications of antibiotic treatment in your patients?
   1 - not at all 2 - slightly
   3 - moderately 4 - very
   5 - extremely
   - diarrhea
   - severe hypersensitivity reaction
   - rash
   - vomiting
   - fungal infection
   - disorders of the intestinal microflora (dysbiosis)
   - interaction with the patient's regular medications
   - deterioration of renal function
   - drug-induced liver damage with an increase in AST/ALT

Patient section

1. In your daily practice, do you encounter attempts by adult patients to force a prescription for an antibiotic for upper respiratory tract infections?
   - Yes
   - No

2. In your daily practice, do you encounter attempts by parents to force a prescription for an antibiotic for a child for upper respiratory tract infections?
   - Yes
   - No

*If "Yes" in question 1 or 2, then move to question 3, if "No" in questions 1 and 2, then skip

question 3.

3. On a scale of 1 to 5, how often do patients report the following reasons for their requests for an antibiotic for themselves or their child?
   1 - never 2 - rarely 3 - sometimes 4 - often 5 - always
   - I know my body, only an antibiotic can help me recover
   - I want/need to recover quickly
   - I am afraid of complications of the infection
   - I have a fever, so I need to get an antibiotic
   - I had similar symptoms recently, and an antibiotic helped me
   - A household member is also sick and received an antibiotic from their doctor
   - I have green nasal discharge
   - I have a sore throat for which nothing helps

4. In your daily practice, do you encounter self-medication with antibiotics by your patients for upper respiratory tract infections?
   - Yes
   - No

5. [If “Yes” in question 2] Who do you think most often uses self-medication with antibiotics for upper respiratory tract infections?
- females - males
- elderly people - young people
- people with higher education - people with primary education
- urban residents - rural residents
- people with higher socioeconomic status - people with lower socioeconomic status

6. On a scale of 1 to 5, how often do you encounter refusal of antibiotic therapy for upper respiratory tract infections among your adult patients?
   1 - never 2 - rarely 3 - sometimes 4 - often 5 - always

7. On a scale of 1 to 5, how often do you encounter refusal of antibiotic therapy for upper respiratory tract infections for a child by parents?
   1 - never 2 - rarely 3 - sometimes 4 - often 5 - always

Knowledge section

1. The first-line antibiotic for non-recurrent streptococcal pharyngitis in a patient without known hypersensitivity reaction is:
   - amoxicillin with clavulanic acid
   - cefuroxime axetil
   - phenoxymethylpenicillin
   - azithromycin
   - clindamycin
   - none of the above

2. The second-line antibiotic for recurrent symptoms of streptococcal pharyngitis or failure of first-line treatment without an identified cause of recurrence is:

- amoxicillin
- cefuroxime axetil
- levofloxacin
- azithromycin
- clindamycin
- none of the above

3. In what situation do you decide to start antibiotic therapy in a child diagnosed with otitis media:
   - immediately after the diagnosis of unilateral acute otitis media in a child under 2 years of age
   - immediately in a child with a high fever above 39˚C and severe pain regardless of age
   - in case of no improvement after 24 hours of anti-inflammatory treatment
   - in case of no improvement after 48-72h of symptoms in a child under 6 months of age
   - none of the above

4. What treatment would you administer to a child presenting at the primary care physician's office with acute bronchitis on the third day of symptoms, in good general condition, without signs of respiratory distress or drops in saturation:
   - inhaled GCS
   - amoxicillin with clavulanic acid
   - levofloxacin
   - inhalations with 3% salt
   - hydration, nasal cleaning, antipyretics, and possible administration of a mucolytic or peripheral cough suppressant depending on the type of cough
   - none of the above

5. What additional test would you order for a patient with bronchial asthma presenting at the primary care physician's office with the following symptoms lasting for 2 days: runny nose, productive cough, fever over 38˚C, headache?
   - rapid CRP test
   - Strep test
   - CBC and CRP from venous blood
   - so-called Combo antigen test (influenza, COVID, RSV)
   - nasal swab culture
   - I wouldn't order any additional tests

Antibiotic resistance awareness section

1. Which of the following antibiotic resistance educational activities in the treatment of upper respiratory tract diseases are you familiar with?
    - The Supreme Medical Board's campaign "Education on the rational use of antibiotics in upper respiratory tract diseases".
    - The article "Rational antibiotic therapy in upper respiratory tract infections - recommendations versus patient perspective. Analysis of the results of the IPSOS 2022 survey".
    - Educational campaign with the "9 out of 10 throat infections are caused by viruses" slogan.
    - Scientific conferences on the topic of antibiotic resistance in the treatment of upper respiratory tract infections.
    - Scientific guidelines on the topic of antibiotic resistance in the treatment of upper respiratory tract infections.
    - Other than the listed activities with the theme of antibiotic resistance in the treatment of upper respiratory tract infections.
    - I am not aware of any activities on antibiotic resistance in the treatment of upper respiratory infections.

2. Would you say that you currently prescribe antibiotics for upper respiratory tract infections:
    - Much more often than 2 years ago
    - Slightly more often than 2 years ago
    - As often as 2 years ago
    - Slightly less often than 2 years ago
    - Much less often than 2 years ago

3. In your opinion, what has influenced the "significant decrease" or "decline" in antibiotic prescribing?
    - The growing number of scientific reports on antibiotic resistance
    - Growing awareness in the medical community about the antibiotic resistance phenomenon
    - Declining availability of antibiotics
    - Fewer patients are now asking for antibiotics
    - Increased access to rapid CRP testing in the primary healthcare setting
    - Increased access to Strep testing within the primary healthcare setting
    - Increased access to the so-called Combo 3-in-1 antigen tests in the primary healthcare setting
    - Educational campaigns aimed at physicians
    - Own improvement of professional competence (training)
    - Increasing number of allergies to antibiotics among patients
    - Growing number of patients refusing to take antibiotics
